# Competition from newborn granule cells does not drive axonal retraction of silenced old granule cells in the adult hippocampus

**DOI:** 10.3389/fncir.2012.00085

**Published:** 2012-11-16

**Authors:** Carla M. Lopez, Kenneth A. Pelkey, Ramesh Chittajallu, Toshiaki Nakashiba, Katalin Tóth, Susumu Tonegawa, Chris J. McBain

**Affiliations:** ^1^Program in Developmental Neurobiology, Eunice Kennedy-Shriver National Institute of Child Health and Human Development, National Institutes of HealthBethesda, MD, USA; ^2^RIKEN-MIT Center for Neural Circuit Genetics and The Picower Institute for Learning and Memory, Massachusetts Institute of TechnologyCambridge, MA, USA; ^3^Department of Psychiatry and Neuroscience, Quebec Mental Health Institute, Université LavalQuebec City, QC, Canada

**Keywords:** neurogenesis, mossy fibers, hippocampal, activity-dependent circuit refinement, tetanus toxin, Moloney virus

## Abstract

In the developing nervous system synaptic refinement, typified by the neuromuscular junction where supernumerary connections are eliminated by axon retraction leaving the postsynaptic target innervated by a single dominant input, critically regulates neuronal circuit formation. Whether such competition-based pruning continues in established circuits of mature animals remains unknown. This question is particularly relevant in the context of adult neurogenesis where newborn cells must integrate into preexisting circuits, and thus, potentially compete with functionally mature synapses to gain access to their postsynaptic targets. The hippocampus plays an important role in memory formation/retrieval and the dentate gyrus (DG) subfield exhibits continued neurogenesis into adulthood. Therefore, this region contains both mature granule cells (old GCs) and immature recently born GCs that are generated throughout adult life (young GCs), providing a neurogenic niche model to examine the role of competition in synaptic refinement. Recent work from an independent group in developing animals indicated that embryonically/early postnatal generated GCs placed at a competitive disadvantage by selective expression of tetanus toxin (TeTX) to prevent synaptic release rapidly retracted their axons, and that this retraction was driven by competition from newborn GCs lacking TeTX. In contrast, following 3–6 months of selective TeTX expression in old GCs of adult mice we did not observe any evidence of axon retraction. Indeed ultrastructural analyses indicated that the terminals of silenced GCs even maintained synaptic contact with their postsynaptic targets. Furthermore, we did not detect any significant differences in the electrophysiological properties between old GCs in control and TeTX conditions. Thus, our data demonstrate a remarkable stability in the face of a relatively prolonged period of altered synaptic competition between two populations of neurons within the adult brain.

## Introduction

The subgranular zone of the hippocampal dentate gyrus (DG) represents one of only two privileged sites within the mammalian central nervous system where adult neurogenesis occurs, providing a continuous source of newborn granule cells (GCs) throughout postnatal life (Deng et al., 2010; Ming and Song, 2011). Newborn GCs initially exhibit molecular, anatomical, and physiological properties distinct from their mature counterparts; however, within approximately 4–8 weeks of maturation newborn GCs appear phenotypically indistinguishable from the preexisting mature GC cohort (Laplagne et al., 2006; Ge et al., 2008). Thus, in adult mice the mature GC population comprises both embryonically/early postnatal generated GCs as well as postnatally derived GCs beyond roughly 2 months of age (hereafter referred to as old GCs). Whereas active adult neurogenesis was once widely regarded as a simple neuronal replacement mechanism, recent studies indicate that newborn GCs make unique contributions to hippocampal-dependent learning and episodic memory. Specifically, newborn GCs critically participate in the formation of distinct memories of similar events by encoding unique representations of the spatial relationships of a given experience. This “pattern separation” function of the hippocampus is essential for discriminating between similar episodic memories with overlapping features and is compromised or enhanced in animals with disrupted or augmented postnatal neurogenesis respectively (Clelland et al., 2009; Creer et al., 2010; Sahay et al., 2011; Nakashiba et al., 2012; Tronel et al., 2012).

**Figure 1:**
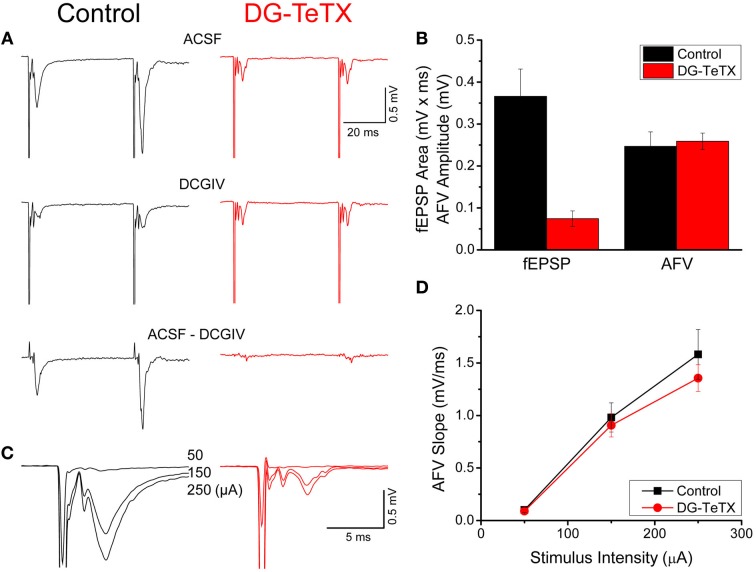
**Impaired MF-CA3 transmission but normal MF excitability in activated DG-TeTX mice. (A)** Representative fEPSP waveforms recorded in CA3 stratum lucidum evoked by stimulation in the DG GC layer obtained in control and activated DG-TeTX (DG-TeTX) mice in control (ACSF, upper traces) and DCGIV (1 μM) containing ACSF (DCGIV, middle traces). Traces are the average of 6 consecutive individual sweeps obtained in each condition (scale bars 20 ms/0.5 mV). The pure MF-CA3-mediated fEPSP is obtained by digital subtraction of the DCGIV condition waveform from the control condition waveform (bottom traces). **(B)** Group data bar chart summary of the MF-CA3 fEPSP areas and AFV amplitudes observed in control (*n* = 10 slices from 7 animals) and DG-TeTX (*n* = 7 slices from 5 animals) mice. **(C)** Representative fEPSP waveforms from control and DG-TeTX mice obtained at the three different stimulus intensities indicated (traces are the average of 6 events obtained at each stimulus intensity, scale bars 5 ms/0.5 mV). **(D)** AFV input-output functions for control (*n* = 8 slices from 5 mice) and activated DG-TeTX mice (*n* = 10 slices, from 5 mice).

To mediate pattern separation newborn GCs must become synaptically integrated into a preexisting hippocampal network. In the developing nervous system synapse formation and refinement are activity-dependent processes and competition between afferents for common postsynaptic targets ultimately dictates mature circuit innervation patterns (Katz and Shatz, 1996; Sanes and Lichtman, 1999; Yu et al., 2004; Hashimoto and Kano, 2005). This raises the possibility that newborn GCs must compete with old GCs to receive afferent input from the entorhinal cortex (EC) and to innervate postsynaptic targets such as CA3 pyramidal cells when integrating into the DG-CA3 circuit (Toni et al., 2007, 2008; Bergami and Berninger, 2012). Consistent with this possibility a study in developing animals (2–4 weeks of age) found that synaptic silencing of old GCs by selective expression of tetanus toxin (TeTX) light chain promoted retraction of their mossy fiber (MF) axons in a manner driven by competition from newborn GCs that continued to transmit to their postsynaptic targets (Yasuda et al., 2011). Moreover, following MF retraction the population of TeTX expressing inactive GCs began to die off beyond postnatal day 25 indicating that competition from newborn GCs may ultimately dictate overall survival of old GCs during the first postnatal month (Yasuda et al., 2011). Whether such competition-based axon refinement and cell survival occurs in the adult DG was not determined. However, in a recent investigation examining the functional roles of old GCs and adult generated newborn GCs in learning and memory we generated a transgenic line of mice that conditionally/inducibly express TeTX selectively in old GCs beyond 4–6 weeks of age (DG-TeTX mice) and did not observe any evidence of cell death or axon retraction in the silenced GCs (Nakashiba et al., 2012). Indeed electrophysiological experiments in tissue from control and activated DG-TeTX adult mice indicated comparable MF axon densities, inconsistent with significant axon retraction and cell death in silenced GCs [(Nakashiba et al., 2012) and see Figure 1]. Moreover, the full recovery of MF transmission in activated DG-TeTX mice after turning-off TeTX expression (i.e., the transgenic strategy employed allows for inducible and fully reversible synaptic silencing) further argues against significant axon retraction and cell death within the synaptically silenced GC cohort (Nakashiba et al., 2012).

Here we specifically examined the impact of prolonged synaptic silencing on the properties of old GCs in adult mice using a combination of electrophysiology, immunocytochemistry, and electron microscopy to compare activated DG-TeTX mice and their control littermates. Our data reveal a remarkable stability in the morphological and electrophysiological properties of old GCs following 3–6 months of synaptic silencing. Thus, in contrast to developing systems, neither the survival of old GCs nor maintenance of their MF projections are dictated by output-based competition with newborn GCs in the adult brain.

## Materials and methods

All procedures relating to animal care and treatment conformed to Institutional and NIH guidelines.

### Generation of mice

DG-TeTX mice maintained in a C57BL/6 genetic background harbor three transgenes; POMC-Cre (TG1), αCamKII-loxP-STOP-loxP-tTA (Tg2), and TetO-TeTX (Tg3-TeTX) as previously described in Nakashiba et al. (2012). To obtain DG-TeTX mice, heterozygous Tg1 × Tg3 – TeTX (*POMC-Cre/+, TetO-TeTX/+*) mice were crossed to generate homozygous double transgenic mice (*POMC-Cre/POMC-Cre, TetO-TeTX/TetO-TeTX*). Male homozygous mice were then bred with the female Tg2 (*αCamKII-loxP-STOP-loxP-tTA/+*). Half of the resultant progeny was thus heterozygous triple-transgenic mice (*POMC-Cre/+, TetO-TeTX/+, αCamKII-loxP-STOP-loxP-tTA/+*), herein referred to as DG-TeTX mice. The other half of the progeny was thus heterozygous double transgenic mice (*POMC-Cre/+, TetO-TeTX/+, +/+*), which did not express TeTX and therefore served as control mice. Breeding pairs and their progeny for experiments were maintained on a Dox diet, and then all experiments were performed on tissue from adult mice (3–8 months old) maintained on a Dox free diet for at least 2 months to induce TeTX expression in DG-TeTX mice.

### Virus generation and injection

We used a Moloney viral vector to express GFP obtained from Dr. Carlos Lois (M.I.T.). Moloney viral particles were produced as previously described in Nakashiba et al. (2012). Viral titers were ~10^9^ infectious units/ml. DG-TeTX mice and their control littermates (12–16 weeks old) were anesthetized with avertin and stereotaxically injected at two sites targeting the right DG (0.9 μl of viral aliquot per site). The stereotaxic coordinate for the first site was 2.06 mm posterior from bregma, 1.25 mm lateral from the midline, and 1.75 mm ventral from the brain surface. The second site was 2.70 mm posterior from bregma, 2.00 mm lateral from the midline, and 1.75 mm ventral from the brain surface. The scalp incision was sutured, and postinjection analgesics were given to aid recovery (1.5 mg/kg, Metacam).

### Immunohistochemistry

For immunohistochemical visualization of the stratum lucidum and adult-born GFP + GCs, mice were perfused transcardially using a 0.1 M PBS solution containing 4% paraformaldehyde followed by overnight postfixation at 4°C. Brains were then cryoprotected in 30% sucrose/PBS at 4°C, sliced to 50 μm thickness using a microtome. After washing in PBS, free-floating sections were blocked for 2 h at room temperature in a PBS/0.5% Triton X-100/1% BSA/10% normal goat serum (NGS) solution before being incubated overnight at 4°C with anti-calbindin (mouse, 1:1000; Sigma), and anti-GFP (chicken, 1:1000, Aves) antibodies. Primary antibodies were diluted in a PBS/0.5% Triton X-100/1% BSA/10% NGS (BGT-PBS). Slices were washed with BGT-PBS before being incubated for 2 h at room temperature with secondary antibodies diluted in BGT-PBS. After washing in PBS, slices were mounted on Superfrost Plus microscope slides (Fisherbrand, Hampton, NH). Secondary antibodies were used in the following concentrations: goat anti-chicken Alexa Fluor 488 and goat anti-mouse Alexa Fluor 555 (1:1000; Invitrogen). Although the transgene construct for the Tg3-TeTX was initially generated as a fusion protein with GFP, we failed to detect any GFP-TeTX fusion protein in the DG-TeTX mice with this immunostaining procedure (data not shown). Thus, signals detected by anti-GFP here are mostly derived from GFP introduced by the Moloney virus. Fluorescent images were captured using a Zeiss LSM 780 inverted confocal microscope (Zeiss, Germany). To quantify the extent of old GC MF projections into CA3 in individual sections we measured the length of a curved line tracing the GFP signal through stratum lucidum toward CA2 from a straight line drawn connecting the ends of the upper and lower blades of the DG (Zhao et al., 2006). A total of 3–12 sections per animal from similar levels of the hippocampus were examined.

### Electron microscopy

Animals used for the electron microscopy were deeply anesthetized and transcardially perfused first with a buffered sodium sulphide solution (12 g Na_2_S.9H_2_O and 12 g NaH_2_PO_4_.H_2_O in 1000 ml of distilled water, pH 7.4; 0.05 M) for 1 min, then with a buffered 3% glutaraldehyde solution in 0.12 M PBS (pH 7.4) for 20 min, and finally with the sodium sulphide solution again for 15 min. Brains were removed from the skull, postfixed in the 3% buffered glutaraldehyde solution for 2 h and sectioned with a Vibratome at 50 μm. Free-floating sections were washed with Tris buffer (pH 7.4) for 5 min periods in order to eliminate adsorbed phosphate ions, which would react with silver ions, causing an unwanted precipitation. Thereafter, sections were placed in the physical developer containing sodium tungstate as protective colloid, hydroquinone as reducing agent, sodium acetate and acetic acid to adjust the pH and silver nitrate for further details see Seress and Gallyas (2000). The process of development was stopped by placing the sections into 1% sodium thiosulfate for 1 min. Next, the sections were washed with Tris buffer for 5 min, then osmificated with 1% OsO_4_ for 1 h, dehydrated, and flat-embedded in Durcupan according to routine electron-microscopic procedure. After microscopic examination, the area of interest was cut, re-embedded, and thin sectioned. The resulting thin sections were then stained with uranyl acetate and lead citrate and imaged using a Tecnai electron microscope.

#### Quantification

Black particles indicating zinc were used to identify MF terminals. Quantification of area measurements was done by an observer who was blinded to the genotype of the samples. Surface area measurements were done manually using Fiji software.

### *In vitro* slice physiology

Hippocampal slices (300 μm thick) were prepared from 3- to 8-month-old DG-TeTX mice and their control littermates. Mice were anesthetized with isoflurane, and brains were dissected in partial sucrose artificial cerebrospinal fluid (ACSF) containing (in mM): 80 NaCl, 3.5 KCl, 1.25 H_2_PO_4_, 25 NaHCO_3_, 4.5 MgSO_4_, 0.5 CaCl_2_, 10 glucose and 90 sucrose, equilibrated with 95% O_2_ and 5% CO_2_. The brains were hemisected, and transverse slices were cut using a VT-1000S vibratome (Leica Microsystems). The slices were then incubated in the above solution at 35°C for 30 min and then kept at room temperature in the same solution until use.

For extracellular field recordings, slices were transferred to a recording chamber and perfused (3–5 ml/min, 32–35°C) with ACSF composed of (in mM): 130 NaCl, 24 NaHCO_3_, 3.5 KCl, 1.25 NaH_2_PO4, 2.5 CaCl_2_, 1.5 MgCl_2_, 10 glucose, 0.05 ± dl-AP5, and 0.01 bicuculline methobromide, saturated with 95% O_2_ and 5% CO_2_, pH 7.4. Field excitatory postsynaptic potentials (fEPSPs) were recorded using electrodes (2–3 MΩ) pulled from borosilicate glass (World Precision Instruments) filled with oxygenated ACSF and connected to a Multiclamp 700A amplifier (Axon Instruments, Foster City, CA). Mossy fiber to CA3 pyramid (MF→CA3) fEPSPs were recorded by placing the recording electrode in stratum lucidum and evoking synaptic responses at 0.1 Hz by stimulation (150-μs duration, 0.05- to 0.25-mA intensity) via a constant-current isolation unit (A360, World Precision Instruments, Sarasota, FL) connected to glass electrode filled with oxygenated ACSF placed in the DG cell layer. Data acquisition (filtered at 3 kHz and digitized at 20 kHz) and analysis were performed using a PC equipped with pClamp 9.2 software (Axon Instruments).

MF afferent fiber volley (AFV) input-output (I/O) relations were obtained by stepping the stimulus intensity from 0.05 mA to 0.25 mA. Then fEPSPs monitored at a single stimulus intensity that gave AFVs of between 0.2 and 0.3 mVs were obtained in control ACSF and again in the presence of DCGIV (2 μM, Tocris). Averaged waveforms (10 consecutive sweeps) obtained in DCGIV were digitally subtracted from the corresponding averaged waveform obtained in control ACSF to obtain pure MF→CA3 fEPSPs (Kamiya et al., 1996). For analysis, the area of the pure MF→CA3 fEPSPs was determined in the first 2.5 ms after the end of the AFV determined prior to the digital subtraction. Area was used rather than peak or slope because MF→CA3 fEPSPs have complicated waveforms that may confound peak or slope measurements, particularly in the activated DG-TeTX mice (Henze et al., 2000; Nakashiba et al., 2012). AFV amplitudes and slopes were measured directly from non-DCGIV-subtracted traces.

To characterize the electrophysiological properties of individual mature GCs we performed whole-cell patch clamp recordings in slices prepared from 4 to 6 month old mice 3–4 weeks after Moloney virus injection targeting GFP negative GCs near the inner molecular layer. Slices were perfused (3–5 ml/min) with extracellular solution composed of (in mM) 130 NaCl, 24 NaHCO_3_, 3.5 KCl, 1.25 NaH_2_PO_4_, 2.5 CaCl_2_, 1.5 MgCl_2_, and 10 glucose, saturated with 95% O_2_ and 5% CO_2_ (pH 7.4). Recordings were performed at 32–34°C with electrodes (3–5 MΩ) pulled from borosilicate glass (World precision instruments) filled with either (in mM) 150 K-gluconate, 3 MgCl_2_, 0.5 EGTA, 2 MgATP, 0.3 Na2GTP, and 10 HEPES plus 2 mg/ml biocytin for characterization of membrane properties and LTP experiments or 130 CsCl, 8.5 NaCl, 0.5 EGTA, 4 MgATP, 0.5 Na2GTP, 5 QX-314Cl, and 10 HEPES for basic postsynaptic current (excitatory and inhibitory) characterization. Whole-cell patch clamp recordings were made using a Multiclamp 700A or 700B amplifier (Molecular Devices, Sunnyvale, CA) in current- or voltage clamp-mode. The signals were filtered at 3 kHz (Bessel filter; Frequency Devices, Haverhill, MA) and digitized at 20 kHz (Digidata 1322A or 1440A and pClamp 9.2 or 10.2 Software; Molecular Devices). Recordings were not corrected for a liquid junction potential.

The resting membrane potential was noted immediately upon achieving a whole-cell configuration. The membrane potential was then biased to −60 mV by constant current injection. The input resistance (*R*_*m*_) was measured using a linear regression of voltage deflections (±15 mV from the resting potential, ~60 mV) in response to 2-s current steps of 6–10 different amplitudes in 5-pA steps. The membrane time constant (Tau_*m*_) was calculated from the mean responses to 20 successive hyperpolarizing current pulses (−20 pA; 400 ms) and was determined by fitting voltage responses with a single exponential function. Action potential (AP) threshold was defined as the voltage at which the slope trajectory reached 10 mV/ms and AP amplitude was defined as the difference in membrane potential between the threshold and the peak. Spike half-width was defined as the duration of the AP at half of the determined amplitude. These properties were measured for the first two APs elicited by a depolarizing 800 ms current pulse of amplitude that was just sufficient to bring the cell to threshold for AP generation. Firing frequency was calculated from the number of spikes observed during the 800 ms window during a current injection twice this amount. All intrinsic electrophysiological parameters were measured in pClamp or using procedures written in Igor 6 (Wavemetrics, Portland, OR).

Spontaneous inhibitory postsynaptic currents (sIPSCs) were pharmacologically isolated by the addition of DNQX (10 μM) and dl-AP5 (50 μM) to the perfusing medium and recorded as inward currents at a holding potential of −70 mV with the chloride reversal potential set to 0 mV (CsCl-based internal). Events were detected using a template matching algorithm and analyzed in pClamp. At the end of the recordings, bicuculline (10 μM) was added to the perfusing medium to determine the tonic GABAergic current. Spontaneous and evoked excitatory postsynaptic currents (s/eEPSCs) were pharmacologically isolated by the addition of bicuculline (10 μM) to the perfusing medium. sEPSCs were recorded at a holding potential of −70 mV and detected by a template matching strategy and analyzed in pClamp. eEPSCs were elicited at 0.1 Hz as paired pulses (20 Hz) by low-intensity microstimulation (100 μs duration; 10–30 μA intensity) via a constant-current isolation unit (A360, World Precision Instruments, Sarasota, FL) connected to a patch electrode filled with oxygenated extracellular solution placed in the middle molecular layer. The AMPA receptor-mediated component was determined from the peak of the eEPSC (the first event of paired pulses) observed at a holding potential of −70 mV, and the NMDA receptor-mediated component was measured 25 ms after the peak at a holding potential of +40 mV. Paired pulse ratios (PPRs) were calculated as the mean P2/mean P1, where P1 was the amplitude of the first evoked current and P2 was the amplitude of the second synaptic current obtained for consecutive individual traces.

For LTP experiments, bicuculline was added to the perfusing medium, and AMPAR-mediated EPSCs were monitored in voltage clamp mode (*V*_*h*_= −70 mV) before (2–3 min) and after (15–20 min) LTP induction with a theta burst stimulation (TBS) protocol (Schmidt-Hieber et al., 2004). TBS was performed in current clamp mode with the membrane potential biased to −60 mV via constant current injection. TBS consisted of 10 trains of stimuli at 5 Hz, with each train consisting of 10 stimuli at 100 Hz. The duration of each train was paired with a postsynaptic depolarizing current injection of 300–400 pA that was sufficient to evoke a burst of postsynaptic APs. This was repeated four times at a frequency of 0.1 Hz, after which the recording configuration was switched back to voltage clamp mode, and AMPAR-mediated EPSCs were monitored as described above for a minimum of 20 min post-TBS.

For all electrophysiological parameters tested each cell was treated as an independent observation. Data are presented as means ± SEMs unless otherwise indicated. Data sets were subjected to a Normality Test and statistical significance was assessed using parametric (Student's *t*-test for normally distributed data sets) or nonparametric (Mann–Whitney test for data sets not normally distributed) analyses as appropriate.

For anatomical reconstruction of recorded cells after biocytin filling during whole-cell recordings, slices were fixed with 4% paraformaldehyde and stored at 4°C then permeabilized with 0.3% Triton X-100 and incubated with Alexa Fluor 555-conjugated streptavidin (Molecular Probes). Resectioned slices were mounted on gelatin-coated slides using Mowiol mounting medium. Cells were visualized using epifluorescence microscopy (Olympus AX70) and images for representative examples were obtained with a Leica TCS SP2 RS Confocal Microcope. Frames of maximum projection images were created from stacks (2 μm steps) and stitched together in Adobe Photoshop.

## Results

To examine the role of activity and competition in regulating hippocampal GC anatomy and function we compared several features of old GCs from adult activated triple transgenic DG-TeTX mice (Nakashiba et al., 2012) with those from control littermate adult mice (Control). In the DG-TeTX mice, TeTX expression is induced selectively and comprehensively in old GCs (beyond 4–6 weeks of age) when the mice are kept on a doxycycline (Dox) free diet, thus, effectively silencing the synaptic output of the vast majority of GCs by preventing presynaptic vesicle fusion (Nakashiba et al., 2012). In contrast, due to the delayed onset of αCamKII promoter activity (see methods) newborn GCs in DG-TeTX mice cannot be induced to express TeTX within the first 4–6 weeks after their genesis (Nakashiba et al., 2012) leaving them release competent and potentially placing them at a competitive advantage within the hippocampal circuit. Consistent with our prior observations, activated DG-TeTX mice exhibited severely impaired MF transmission as revealed by an almost complete loss of MF-CA3 fEPSPs in DG-TeTX mice kept on a Dox free diet for 2–3 months (Figures 1A,B). It is important to note that although young GCs remain release competent their potential contribution to the MF-CA3 fEPSP is minimal due to their low numbers [<5% of the entire GC population, (Imayoshi et al., 2008)] and the sparse nature of GC release sites (Amaral and Dent, 1981; Claiborne et al., 1993; Henze et al., 2000). Despite deficiencies in the fEPSPs of activated DG-TeTX mice, MF excitability was comparable to control animals as indicated by examination of the MF AFVs (Figure 1B). Indeed I/O functions of AFVs in activated DG-TeTX mice revealed no significant differences with those obtained from control mice (Figures 1C,D). Because the AFV reflects the simultaneous activation of multiple tightly packed axons near the recording electrode its magnitude serves as a proxy measure for the density of MF axons within stratum lucidum where the recordings are made. While our experiments do not allow us to determine an absolute value of fiber density the comparable I/O curves obtained in control and DG-TeTX mice indicate similar MF densities providing initial evidence that old GCs silenced in activated DG-TeTX mice remain healthy and do not retract their MF axons from stratum lucidum.

**Figure 2:**
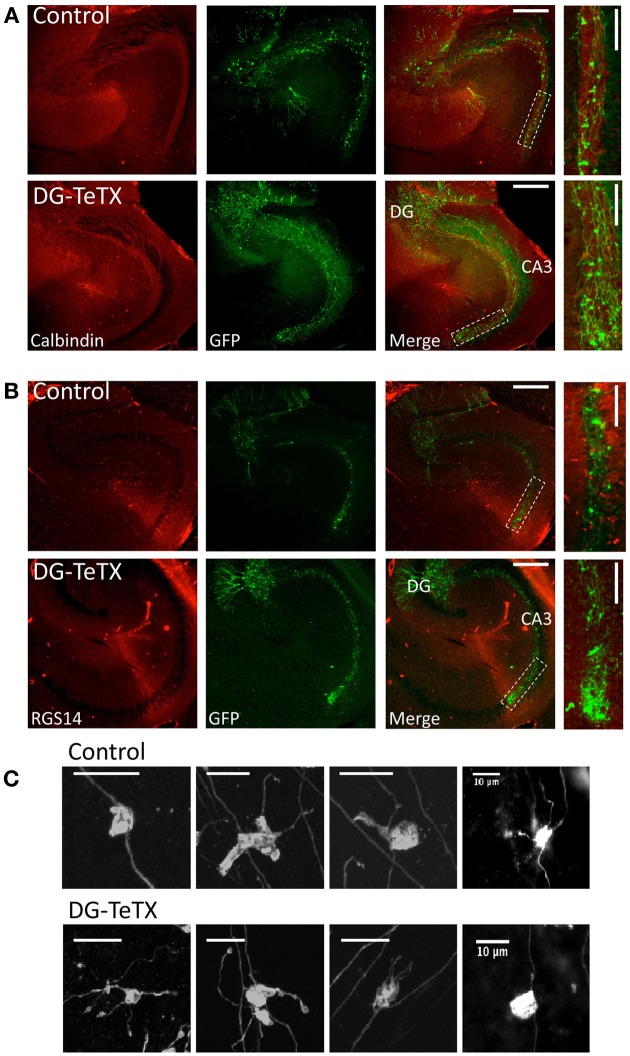
**Normal MF projection throughout CA3 stratum lucidum in activated DG-TeTX mice. (A)** Representative images of hippocampal sections obtained from control (upper panels) and activated DG-TeTX mice (lower panels) stained for calbindin (red) to label stratum lucidum and GFP (green) to label silenced old GCs (at least 3 months of age) that were infected with GFP encoding Moloney Virus at their time of genesis (scale bars 300 μm). Boxed region in the merged images indicate regions that were digitally magnified in images at the far right (scale bars 100 μm). **(B)** Similar to **(A)** except that the sections were stained for GFP (green) and RGS14 (red) to label CA2 pyramidal cells demarcating the distal boundary of CA3. **(C)** Images of representative GFP labeled MFBs from old GCs in sections from control (upper panels) and activated DG-TeTX mice (lower panels). Similar observations were obtained in a total of 5 mutant and 4 control mice examined 3–6 months after viral infection and maintained under Dox free conditions for this entire period (mutant mice were examined at 100, 127, 182, 182, and 178 days postinfection and control mice were examined in parallel at 100, 127, 189, and 162 days postinfection).

Next we probed for evidence of competition-based MF retraction by examining individually labeled axons of silenced GCs. To label individual GCs we injected a genetically modified Moloney viral vector encoding GFP into the DG of control and activated DG-TeTX mice to specifically infect a small cohort of newborn GCs generated at or shortly after the time of viral infection (Van Praag et al., 2002; Tashiro et al., 2006; Zhao et al., 2006). Immediately following viral injection the animals were placed on a Dox free diet and allowed to survive for an additional 3–6 months providing sufficient time for the labeled cohort of cells to enter the old GC population and become silenced in the mutant mice by induction of TeTX as the cells aged beyond 4–6 weeks. Individual GFP labeled MFs were found to project into CA3 stratum lucidum in both control and activated DG-TeTX mice with no obvious signs of axon retraction in the silenced fibers (Figure 2). Indeed in both lines of mice GFP labeled fibers with regularly spaced MF boutons (MFBs) coursed through the entire CA3 stratum lucidum region, revealed by co-staining for calbindin (Figure 2A), terminating at the distal junction with CA2 revealed by co-staining for the CA2 pyramidal cell marker RGS14 [Figure 2B; (Lee et al., 2010)]. Quantification of the extent of old GC MF projections into CA3 by measuring the distance from the hilar border to the farthest projections of GFP labeled fibers (Zhao et al., 2006) revealed no significant differences between control (1220 ± 31 μm, *n* = 4 mice) and activated DG-TeTX mice (1271 ± 51 μm, *n* = 5 mice; *p* = 0.4, *t*-test). Upon closer inspection at higher magnification typical MFBs with associated filopodial extensions were evident (Figure 2C) in control and mutant animals, but we found no evidence of axon degeneration such as retraction bulbs or axosomes that were previously reported for silenced MFs in young developing mice (Yasuda et al., 2011).

**Figure 3:**
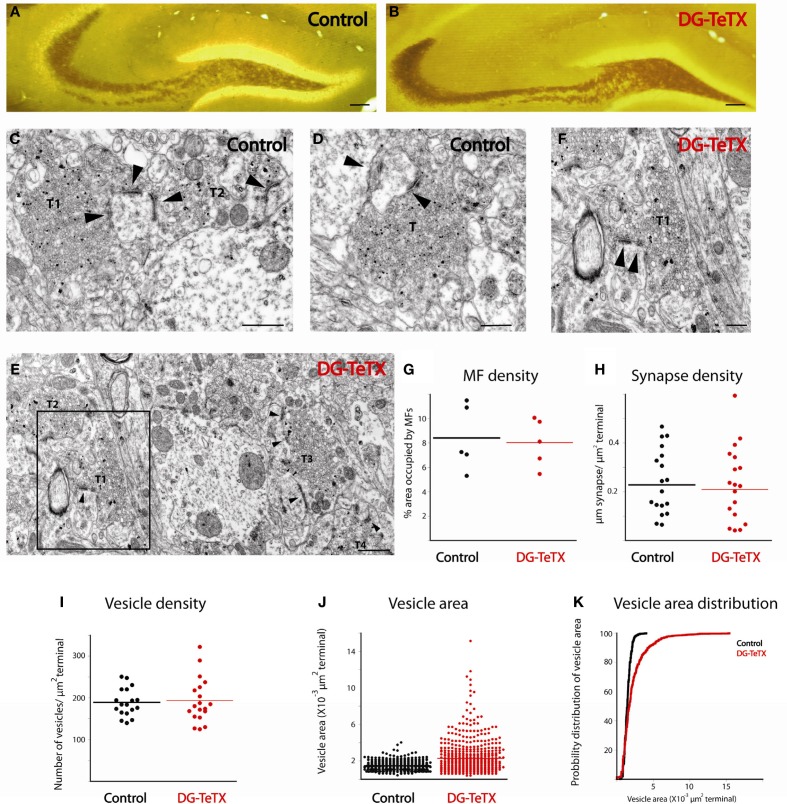
**Ultrastructural comparison of active and silenced MF terminals. (A,B)** Mossy fiber tracks in representative sections from control **(A)** and DG-TeTX **(B)** mice are labeled with neoTimm staining. **(C–F)** Representative electron micrographs demonstrate that mossy fiber terminals (T) form multiple synaptic contacts (arrowheads) with their postsynaptic targets in control **(C,D)** and in DG-TeTX **(E,F)** mice. Zinc-labeled terminals (T1–T3) are abundant in the stratum lucidum of DG-TeTX samples **(E)**, T1 is shown in panel F at higher magnification where the high degree of variability in vesicle size is clearly evident. **(G)** The density of mossy fibers measured as a ratio of the percentage of surface area covered by zinc-positive large presynaptic specializations was similar in the stratum lucidum of control and DG-TeTX animals (*n* = 5 sections per animal, 1 mouse per genotype). **(H,I)** Synapse density (**H**, *n* = 20 terminals examined across the 5 sections for each genotype) and vesicle density (**I**, *n* = 20 terminals examined across the 5 sections for each genotype) was also comparable between the two groups. **(J)** Plots of vesicle area reveal a typical uniform vesicle size in control samples but a higher degree of variability in DG-TeTX mice. **(K)** Cumulative probability distribution of vesicle size measurements plotted in **(J)** revealing the skew toward larger vesicles in DG-TeTX mice (*n* = 620 vesicles in 20 terminals across 5 sections from 1 mouse per genotype). Scale bars: (**A,B**: 200 μm, **C–E**: 400 nm, **F**: 800 nm).

The persistence of MFBs along the axons of old GCs in activated DG-TeTX mice suggests that the silenced terminals maintain structural contact with postsynaptic targets despite their lack of vesicle-mediated transmission consistent with the ability to recover MF-CA3 transmission when DG-TeTX mice are re-treated with Dox (Nakashiba et al., 2012). To examine the effects of synaptic silencing on the ultrastructural features of MFBs and associated postsynaptic elements we compared terminals in control and activated DG-TeTX mice using electron microscopy. To easily identify MFBs, sections were neoTimm stained to reveal the zinc rich MF projections (Haug, 1967; Ibata and Otsuka, 1969; Amaral and Dent, 1981). Importantly, at the light microscopy level a strong zinc signal was clearly evident throughout the entire stratum lucidum in hippocampal sections from both control and activated DG-TeTX mice consistent with maintained MF projections to distal CA3 in silenced GCs (Figures 3A,B). At the EM level typical zinc labeled MF terminals with multiple release sites and associated postsynaptic densities were readily encountered in tissue from both control and activated DG-TeTX mice with no evidence of axon retraction or degeneration (Figures 3C–F). As expected from our analyses of AFVs (Figures 1B–D), MF density in randomly selected areas of the stratum lucidum was similar in control and activated DG-TeTX mice with ~10% of the total surface identified as MF terminals in both samples (Figure 3G; 8.4 ± 1.2% in control; 8 ± 0.87% in DG-TeTX samples). Moreover, the density of MF synaptic contacts, calculated from the total length of synaptic specializations formed by a single terminal and the surface area of the bouton, was comparable between control and DG-TeTX mice suggesting that silenced terminals maintain a physical interaction with target postsynaptic elements (Figure 3H; 0.22 ± 0.03 μm synapse/μm^2^ terminal and 0.2 ± 0.03 μm synapse/μm^2^ terminal in control and DG-TeTX samples, respectively). Surprisingly, despite the deficiency in VAMP2-mediated release, vesicle density in terminals of DG-TeTX mice was similar to that in control terminals (Figure 3I; 189 ± 8 vesicles/μm^2^ in control and 193 ± 12 vesicles/μm^2^ in DG-TeTX samples). Closer examination of synaptic vesicles revealed that while vesicle density was similar between the two genotypes, vesicle size distributions were not (Figures 3J,K). In control tissue vesicle size showed little variability, and the average vesicle area was 1.47 × 10^−3^ ± 2.02 × 10^−5^ μm^2^ (corresponding to 42 nm vesicle diameter). In DG-TeTX samples the distribution of vesicle size was larger with a noticeable skew toward larger vesicles (2.24 × 10^−3^ ± 7.22 × 10^−5^ μm^2^). Although the limited number of mice examined at the ultrastuctural level precludes statistical comparisons between the genotypes our observations generally confirm that MF axons of old silenced GCs remain stable projecting throughout CA3 stratum lucidum. Moreover, the maintenance of an anatomically defined synaptic contact by silenced terminals in activated DG-TeTX mice suggests that the recovery of transmission upon reversal of TeTX expression by treatment with Dox does not reflect a massive rewiring of the circuit but rather a simple resumption of communication at preexisting connections.

**Figure 4:**
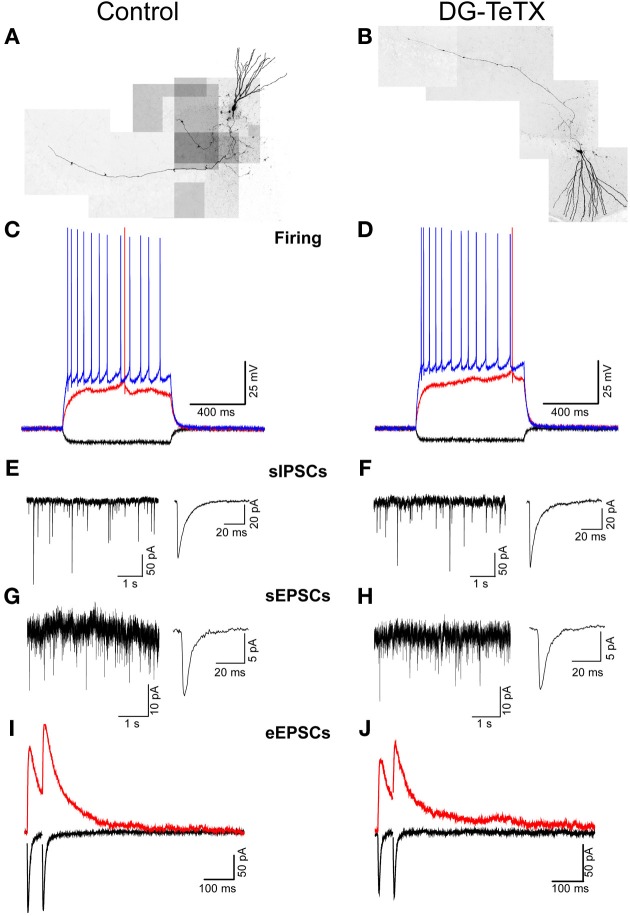
**Prolonged blockade of the synaptic output from old GCs does not alter their electrophysiological characteristics. (A,B)** Composite images of representative old GCs recorded in slices from control **(A)** and activated DG-TeTX **(B)** mice. **(C,D)** Traces from representative recordings obtained in old GCs of control **(C)** and activated DG-TeTX mice **(D)** showing membrane responses to hyperpolarizing (−50 pA; black traces), just threshold level depolarizing (red traces) and twice threshold level depolarizing (blue traces) current pulses. **(E–H)** Representative gap-free traces of pharmacologically isolated spontaneous GABA_A_ receptor- and AMPA receptor-mediated IPSCs **(E,F)** and EPSCs **(G,H)** with ensemble averages (right traces) obtained in old GCs of control **(E,G)** and DG-TeTX **(F,H)** mice. **(I,J)** Paired pulse (50 ms inter-stimulus interval) perforant path-evoked EPSCs at a holding potential of −70 mV (black traces, to monitor the AMPAR-mediated component) and at a holding potential of +40 mV (red traces, to reveal the NMDAR-mediated component) in representative recordings from old GCs in control **(I)** and DG-TeTX **(J)** mice.

**Figure 5:**
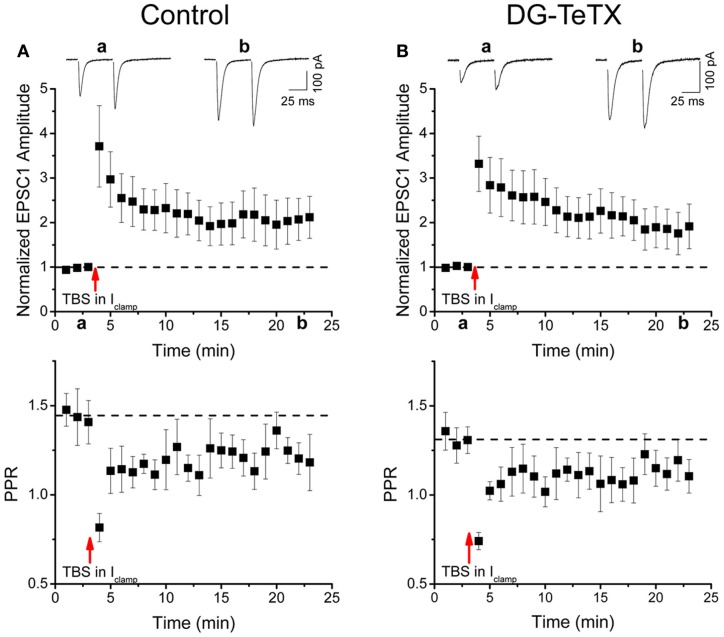
**Normal LTP of perforant path inputs to old GCs in activated DG-TeTX mice. (A,B)** The upper panels are pooled data time course plots showing LTP of perforant path-evoked AMPA receptor-mediated EPSCs in response to a theta burst induction protocol in old GCs of control (**A**, *n* = 5 cells from 3 animals) and activated DG-TeTX (**B**, *n* = 6 cells from 4 animals) mice. Inset traces show EPSCs taken at the times indicated on the *x*-axis. Lower panels show the corresponding pooled data time course plots of the paired pulse ratios for AMPA receptor-mediated EPSC peak amplitudes (PPRS) in control and activated DG-TeTX mice.

Our data to this point indicate that TeTX expressing GCs maintain stable electrically active MF projections throughout the DG-CA3 circuit. However, it remains possible that the silenced old GCs undergo other homeostatically induced changes due to altered participation within the circuit (Desai, 2003; Davis, 2006; Turrigiano, 2012). In our previous study we did not detect any differences in the electrophysiological properties of young (3–4 week old) adult generated GCs in control and activated DG-TeTX mice (Nakashiba et al., 2012). In both lines of mice the newborn cells exhibited immature intrinsic membrane properties and excitatory/inhibitory postsynaptic response profiles typical for cells of their age indicating that being placed at a competitive advantage in activated DG-TeTX mice did not promote enhanced maturation of these physiological properties of adult generated GCs (Nakashiba et al., 2012). However, whether any electrophysiological changes occurred in the synaptically silenced old GCs was not previously tested. Thus, in a final series of experiments we performed whole-cell patch clamp recordings targeting old GCs in acute hippocampal slices from 4–6 month old control and activated DG-TeTX mice to determine if long-term silencing of synaptic output precipitates any changes in basic membrane or postsynaptic properties of GCs. No significant differences were observed between old GCs from the two genotypes for any of the basic membrane and spiking properties assayed including resting membrane potential, input resistance, membrane time constant, and spike frequency/duration (Figures 4A–D and Table 1, *p*-values ranged from 0.1 to 1.0). In addition silenced and control GCs received similar levels of excitatory and inhibitory drive as assessed by monitoring spontaneous glutamatergic and GABAergic synaptic events as well as tonic GABAergic inhibition (Figures 4E–H and Table 1). Comparison of control and activated DG-TeTX old GCs revealed no differences in any of the synaptic properties assayed including inhibitory/excitatory postsynaptic current kinetics, AMPA/NMDA ratios, and short-term synaptic plasticity at perforant path inputs (Figures 4I,J and Table 1, *p*-values ranged 0.2–0.9). Moreover, silenced GCs in activated DG-TeTX mice exhibited theta-burst stimulation-induced long-term potentiation (LTP) of excitatory synaptic transmission that was comparable to active GCs in control mice (Figures 5A,B and Table 1, *p* = 0.8). In both genotypes LTP was associated with a reduction in PPR consistent with an increase in presynaptic function during the expression of this plasticity (Figures 5A,B). Together, these findings indicate that prolonged blockade of synaptic output from old GCs does not precipitate overt changes in their basic electrophysiological and synaptic input properties arguing against any significant homeostatically induced plasticity in the cells due to altered network participation.

**Table 1:** **Summary of electrophysiological properties of mature granule cells**.

	**Control mice**	**DG-TeTX mice**
**Intrinsic membrane properties**	**(*n* = 14 cells from 5 mice)**	**(*n* = 15 cells from 4 mice)**
Resting potential (mV)	−71 ± 1	−68 ± 2
Input resistance (MΩ)	199 ± 14	225 ± 27
Time constant (ms)	23 ± 2	19 ± 2
Membrane capacitance (pF)	117 ± 9	94 ± 8
Frequency at 2× threshold (Hz)	23 ± 2	19 ± 2
Spike threshold (mV)	−35 ± 2	−35 ± 2
Spike amplitude (mV)	77 ± 3	74 ± 3
Spike half-width (ms)	0.84 ± 0.04	0.87 ± 0.06
Spike maximal decay (mV/ms)	−93 ± 5	−90 ± 6
**IPSC properties**	**(*n* = 8 cells from 3 mice)**	**(*n* = 3 cells from 2 mice)**
sIPSC amplitude (pA)	−60 ± 5	−64 ± 14
sIPSC frequency (Hz)	5 ± 0.4	4.3 ± 1.1
sIPSC τ_decay_ (ms)	7.6 ± 0.4	8.2 ± 0.5
GABA_tonic_ (pA)	−30 ± 8	−45 ± 19
**EPSC properties**	**(*n* = 6 cells s/eEPSCs, 5 cells LTP from 3 mice)**	**(*n* = 6 cells s/eEPSCs, 6 cells LTP from 4 mice)**
sEPSC amplitude (pA)	−11 ± 1	−10 ± 0.6
sEPSC frequency (Hz)	1.6 ± 0.3	1.3 ± 0.2
sEPSC τ_decay_ (ms)	5.6 ± 0.5	5.8 ± 0.3
eEPSC AMPA/NMDA ratio	1.3 ± 0.2	1.5 ± 0.2
eEPSC PPR	1.0 ± 0.04	1.2 ± 0.09
eEPSC_NMDA τ_decay__ (ms)	73 ± 3	83 ± 7
eEPSC TBS LTP (% control)	212 ± 47	191 ± 50

## Discussion

Neuronal cell replacement strategies offer incredible therapeutic potential for circuit repair in neurodegenerative disorders or following traumatic brain injury (Lopez-Bendito and Arlotta, 2012). However, the realization of this potential requires a thorough understanding of the cellular mechanisms underlying the integration of new neurons into mature existing neural networks. A wealth of data supports a role for active competition between presynaptic terminals of individual neurons in establishing and sculpting network connectivity in the developing nervous system (Katz and Shatz, 1996; Sanes and Lichtman, 1999; Yu et al., 2004; Hashimoto and Kano, 2005), but whether the circuit integration of new neurons into preexisting adult circuits relies upon similar competition-based axonal refinement is unclear. In a previous study examining the role of young and old GCs in DG function we did not obtain any evidence for altered maturation or circuit integration of newborn GCs in activated DG-TeTX adult mice (Nakashiba et al., 2012). In this model the newborn GCs should be at a competitive advantage in terms of communicating with downstream DG and CA3 postsynaptic targets compared to old GCs that are prevented from releasing transmitter due to the selective expression of TeTX. In the present study we utilized the same neuronal competition model focusing on the silenced old GC population to determine if being placed at a competitive disadvantage within the circuit precipitated any changes in their MF projections or electrophysiological properties. In developing sensory circuits, cerebellum, and neuromuscular junctions neurons placed at such a competitive disadvantage retract their axons following destabilization of their presynaptic terminals triggered by competitive interactions with more active axons innervating the same postsynaptic targets (Fladby and Jansen, 1990; Antonini and Stryker, 1993; Buffelli et al., 2003; Ruthazer et al., 2003; Yu et al., 2004; Hashimoto and Kano, 2005; Hua et al., 2005). In contrast we found no evidence of axon retraction in silenced old GCs of adult DG-TeTX mice. Indeed our electrophysiological, immunocytochemical, and EM data all indicated that the MF axons of presynaptically silenced GCs continue to project throughout the entire extent of CA3 despite potential competitive influences from continuously generated newborn GCs. The silenced axons even retained typical presynaptic specializations, the giant MFBs, in association with postsynaptic targets in stratum lucidum suggesting that VAMP2-dependent release is dispensable in the maintenance of the structural integrity of established MF synapses in adult mice. One caveat to this interpretation of our EM data is that we could not distinguish between terminals of old silenced GCs versus those belonging to newborn GCs leaving open the possibility that our failure to observe terminal changes at the EM level in DG-TeTX mice simply reflects preferential examination of unsilenced terminals from newborn GCs. However, since DG-TeTX mice do not exhibit enhanced neurogenesis (Nakashiba et al., 2012) and newborn GCs comprise only a small proportion of the overall GC population, the similar densities of MFBs in control and DG-TeTX mice argues that our data sets are overwhelmingly comprised of terminals from old silenced GCs in mice of both genotypes. Finally, we found no differences in the basic electrical and synaptic input properties of active and silenced GCs indicating that altered circuit participation of old GCs in activated DG-TeTX mice did not promote homeostatic plasticity of cell membrane properties or excitatory/inhibitory synaptic input to these cells. Together these findings reveal a remarkable stability in the morphological and electrical properties of old GCs following prolonged periods of synaptic silencing.

Our findings contrast starkly with observations in the developing hippocampus of juvenile mice. Using a similar TeTX-based strategy, but with mutants that express TeTX from early postnatal stages, Yasuda et al. (2011) found that silenced GCs undergo axon retraction and elimination in mice 3–4 weeks of age. The TeTX expressing GCs initially projected normally into CA3 within the first two weeks postnatally but then retracted their axons during the 3rd and 4th postnatal weeks and ultimately died after axon elimination. Importantly, this process was prevented by pharmacological global inhibition of circuit activity or suppression of neurogenesis beyond the second postnatal week indicating that axon elimination and cell death of the silenced GCs was driven by activity-dependent competition from newborn GCs that did not express TeTX (Yasuda et al., 2011). Interestingly, in the same study the authors reported a similar competition-based axon elimination in juvenile mice for afferents from the EC and for CA1 pyramidal cell axons (Yasuda et al., 2011). In both cases when TeTX expression was limited to approximately half of the EC and CA1 fibers the authors found an activity-dependent elimination of TeTX expressing axons, presumably driven by the remaining release competent EC and CA1 fibers. Our failure to observe such competition driven axon refinement in the adult hippocampus suggests that this process is limited to a critical period during the first month of hippocampal development or potentially the first 4 weeks postgenesis of an adult born GC. In our model GCs cannot be induced to express TeTX until approximately 6 weeks of age, suggesting that sometime between 3 and 6 weeks of age the synaptic connections made by a GC become immune to any destabilizing influence from more active neighboring fibers that would disrupt the integrity of less active axons and associated terminals. Alternatively, it is possible that by 6 weeks of age the relative contribution of newborn GCs to the entire MF pathway is too small to drive competition-based retraction of large numbers of old GC axons. Such limited competition may only promote axon retraction of a small percentage of silenced old GCs below the detection limits of our experimental approaches. While future experiments designed to increase the degree of competition by enhancing neurogenesis or reducing the percentage of TeTX expressing cells would be informative, our current findings clearly indicate that under basal conditions the competition driven by newborn GCs does not promote gross axon retraction of the entire silenced old GC population in adult mice. Despite the structural immunity to competition observed in silenced GCs, it remains possible that young and old GCs still compete for synaptic control over downstream postsynaptic targets in adult mice. For instance a recent investigation in slice culture indicates that differences in the activity levels among neighboring synapses regulates MFB motility, suggesting that synaptic remodeling events may be influenced by activity-dependent competition (Chierzi et al., 2012). Alternatively, competing inputs to common postsynaptic targets may influence the efficacy of existing synapses without promoting axon retraction by triggering changes in presynaptic release or postsynaptic responsiveness. Future investigations will be necessary to probe for such heterosynaptic plasticity interactions between young and old GCs and may provide insight into the cellular mechanisms underlying the distinct circuit functions of these cohorts in mediating pattern separation and completion.

### Conflict of interest statement

The authors declare that the research was conducted in the absence of any commercial or financial relationships that could be construed as a potential conflict of interest.
